# Clinical and Microbiological Profile of Pathogens in Febrile Neutropenia in Hematological Malignancies: A Single Center Prospective Analysis

**DOI:** 10.1155/2015/596504

**Published:** 2015-07-28

**Authors:** M. Taj, T. Farzana, T. Shah, S. Maqsood, S. S. Ahmed, T. S. Shamsi

**Affiliations:** Department of Clinical Hematology and Bone Marrow Transplantation, National Institute of Blood Disease & Bone Marrow Transplantation, ST 2/A Block 17 Gulshan-e-Iqbal, Sir Shah Suleman Road, KDA Scheme 24, Karachi 75300, Pakistan

## Abstract

*Background*. Febrile neutropenia is the consequence of treatment of hematological disorders. The first-line empirical treatment should cover the prevalent microorganism of the institute. The aim of study was to establish the effectiveness of current practices used at the institution and to review the culture sensitivity pattern of isolated microorganisms. *Patients and Methods*. Data was recorded and analyzed prospectively for 226 hospitalized patients of febrile neutropenia from January 2011 till December 2013. *Results*. Out of 226 cases, 173 were males and 53 were females. Clinically documented infections were 104 (46.01%) and microbiologically documented infections were 80 (35.39%), while 42 (18.58%) had pyrexia of undetermined origin. Gram negative infections accounted for 68 (85%) and *Escherichia coli* was the commonest isolate. Gram positive microorganisms were isolated in 12 (15%) cases and most common was *Staphylococcus aureus*. First-line empirical treatment with piperacillin/tazobactam and amikacin showed response in 184 patients (85.9%) till 72 hours. *Conclusion*. There is marked decline in infections due to Gram positive microorganisms; however, Gram negative infections are still of great concern and need further surveillance. In this study the antibiogram has shown its sensitivity for empirical antibiotic therapy used; hence, it supports continuation of the same practice.

## 1. Introduction

Aggressive chemotherapies have improved the survival of patients with hematological disorders, exposing patients to the risk of bacteremia and sepsis, which is a major cause of morbidity and mortality [1, 2]. Febrile neutropenia is a hematological emergency which develops as a result of treatment of hematological malignancies. The literature shows that life-threatening infection is observed in 48 to 60% of patients with febrile neutropenia [3].

A considerable change has been observed in the pattern susceptibility of the offending organism with the passage of time. For deciding the first-line antibiotic cover in febrile neutropenia, it is deemed necessary to consider the culture and susceptibility pattern of isolated microorganism in that institution [4, 5].

We performed a prospective trial to document the source of infection by culture and susceptibility pattern in patients with febrile neutropenia, so that first-line antibiotic policy can be reviewed with reassurance of better surveillance in the treatment paradigm of hematological malignancies.

## 2. Materials and Patients

Prospective data was collected for the patients who received chemotherapy for the hematological malignancies at National Institute of Blood Disease Center and Bone Marrow Transplantation from November 2011 till December 2013 and developed neutropenia (<500/mm^3^) and fever ≥ 100°F. Moreover, set of cultures were sent (peripheral blood, throat, urine, central line, and any other suspicious site if present) and first-line empirical antibiotics were instituted as piperacillin/tazobactam 4.5 G intravenously every 8 hours and amikacin 15 mg/kg body weight in two divided doses. Patients were excluded from the study if they had known hypersensitivity to any of the prescribed antibiotics in the febrile neutropenia protocol or if they were already on quinolone prophylaxis. Primary diagnoses were acute myeloid leukemia, acute lymphoid leukemia, aplastic anemia, and so forth. For persistent high grade spikes repeat cultures were sent and treatment was changed for persistent fever after 48 hours. The second-line treatment consisted of carbapenem group while amikacin was continued the same way. Blood cultures were processed using the BACTEC blood culture system. Organisms were identified according to routine bacteriological procedures. Antibiotic susceptibility testing was interpreted by disc diffusion method. Results were interpreted according to the Clinical and Laboratory Standards Institute's criteria.

The observations were recorded on special Performa designed for the study from the day of first spike of fever till recovery of absolute neutrophil count (ANC) on discharge from hospital or death of the subject. Parameters analyzed included age, sex, presenting complaints, duration of neutropenia, duration of hospital stay, isolation of microorganism, and their culture susceptibility pattern. Clinically documented infections (CDI) were defined as fever associated with local inflammation, for example, pneumonia, skin infection, or cellulitis, whose microbiological pathogenesis cannot be proven or which cannot be examined. Microbiologically documented infections (MDI) defined as infectious organism detected in blood cultures, even without localized inflammation (no clinical focus) or localized, microbiologically documented, infection, with, or without positive blood cultures. Fever of unknown origin (FUO) is defined as isolated fever, no inflammation evocative for clinical infection and no microbial documentation of the source [1, 2].

Data was described as mean and continuous variables were compared using the independent-samples test while categorical variables were compared using the chi-square test or Fisher's exact test for association. Differences were considered statistically significant when *P* < 0.05. Data were analyzed by statistical software (SPSS for Windows 17.0; SPSS, Chicago, Illinois).

## 3. Results

Total 226 episodes were recorded during 26-month time period and out of them 214 cases were analyzed as they were the cases in which febrile neutropenia treatment was started with piperacillin/tazobactam and amikacin as first-line empirical therapy.  Table 1 shows the baseline characteristics of these patients. The mean age of study population was 28.57 years while median was 20. Most frequent signs and symptoms at presentation were fever and generalized weakness followed by gastrointestinal and respiratory symptoms in systemic review. Mean hemoglobin was 9.3, mean absolute neutrophil count (ANC) was 0.96, and mean platelet count was 56 at the time of presentation. The most frequent hematological disease documented was ALL, 91 cases (40.3%), followed by AML, 45 cases (19.9%), and aplastic anemia, 32 cases (14.2%). Central line catheter was inserted in 72 patients. Granulocyte colony stimulating factor was used in 37 patients (16.3%) and was started on day 7 after chemotherapy. Majority of cases developed neutropenia at the induction phase of chemotherapy in acute leukemia. Specific chemotherapy protocols were used in 167 patients and regimens included UKALL protocol 52 (31.1%), high doses Ara-C in 34 (20%), AML induction in 18 (11%), APML treatment in 11 (6.5%), antithymocyte globulin in 14 (8.3%), and other treatments in 38 (22.7%) patients.

Total clinically documented infections (CDI) were 104 (46.01%), and microbiologically documented infections (MDI) were 80 (35.39%), while in 42 cases (18.54%) no cause of fever could be established (pyrexia of undetermined origin, PUO).

Overall 80 bacterial isolates were documented during the study period and the highest topography reported was blood stream infection 35 (43.75%). Yield from other sites was reported as throat 12 (15%), urine 11 (13.75%), central line 7 (8.75%), and others 9 (11.25%).

Among 80 cultures, there were 68 (85%) Gram negative organisms while Gram positive were only 12 (15%). Gram negative cultures included 27* Escherichia coli* (40%), 12* Klebsiella pneumonia* (17.6%), 8* Klebsiella* spp. (12%), 10* Pseudomonas aeruginosa* (14.7%), and 8* Pseudomonas* spp. (12%). Gram positive isolates had 8* Staphylococcus aureus* (66%) out of which 4 were* Methicillin resistant Staphylococcus aureus (MRSA)* and 2 were* Methicillin sensitive Staphylococcus aureus (MSSA)*, however 02* Vancomycin resistant Enterococci (VRE)* were also isolated.

Table 2 shows sensitivity and resistance pattern of the isolated microorganisms documented. The* Escherichia coli* strains were sensitive to piperacillin/tazobactam (48%), amikacin (88%), and carbapenem group (66.6%) while it was exhibiting very good sensitivity to cefoperazone/sulbactam (96%).* Klebsiella pneumonia* was 41% sensitive to piperacillin/tazobactam and 50% to amikacin.* Klebsiella* species was 87% sensitive to both piperacillin/tazobactam and amikacin*. Pseudomonas aeruginosa* was 100% sensitive to piperacillin/tazobactam and amikacin while* Pseudomonas* species was 75% sensitive to piperacillin/tazobactam and 50% to amikacin. Among Gram positive isolates MRSA was 100% sensitive to vancomycin; however, 02 cases of* vancomycin resistant enterococci* were documented. Other routine Gram negative microorganisms were showing routine sensitivity pattern.

Empirical first-line antibiotics used were piperacillin/tazobactam and amikacin in 214 patients while the rest had other combinations so they were excluded from the study. Treatment was unchanged for first 72 hours if patient became afebrile with hemodynamic improvement; however, for persistent fever spikes 20 patients were shifted to second-line empirical treatment (carbapenem and amikacin). Hence, fever was responsive to first-line antibiotics in 184 patients (85.9%). Median duration of neutropenia was 6 days while median hospital stay was 7.41 days. Among 226 patients 7 patients expired (3.09%), among them 2 had septicemia with hepatic failure, 2 patients had septicemia with multiorgan failure, 1 had septicemia with intra-abdominal bleeding, 1 had intracranial bleeding, and 1 patient had septicemia and developed acute cardiac event. Among these 7 patients positive culture was documented in only 2 patients, one had carbapenem resistant* Escherichia coli,* and another had MRSA while in the rest 5 patients no isolate was identified.

## 4. Discussion

The study demonstrates that Gram negative organisms still predominate in neutropenic patients; however, there is significant fall in frequency of Gram positive infections when compared to past data of same institute. The finding is in conjunction with many other international studies which report* E. coli* as still the most frequently isolated Gram negative organism, also seen in this data [6–8]. Same finding has also been supported by the studies reported from India, Turkey, and Brazil [9–11]. The observations helped selecting empirical antibiotic treatment in febrile neutropenia. In this study also first-line antibiotic therapy was piperacillin/tazobactam and amikacin, which is still the choice because of double Gram negative cover, also showing appropriate sensitivity pattern. This combination is able to cover on average 80% of* Escherichia coli* strains and has been given edge in the literature also [12].

The institutional data firstly published in 2003 had series of 120 febrile neutropenia episodes, with MDI of 50%, out of which 56% were Gram negative isolates constituting predominantly* E. coli and Klebsiella* [13]. At that time we had 43% of Gram positive isolates with* Staphylococcus aureus* being highly prevalent. In 2006 almost same percentages of MDI were reported; however, MDI was only 29.8%, and* Klebsiella* and* Staphylococcus aureus* were the most frequent isolates [14]. In third case series the Gram positive and Gram negative microorganisms were almost equally documented; however, current data had significant drop in Gram positive infections while Gram negative microorganisms showed continued rising trend [15]. Microbiologically documented infections accounted for 35.39% showing a better yield than that documented in the literature [9, 11, 12]. The commonest Gram positive isolate is* Staph aureus* and 4 cases of MRSA were isolated: all were sensitive to vancomycin; however, 02 cases of vancomycin resistant enterococci (VRE) were documented and were treated successfully with linezolid.

In one of the recent studies from Iran 67% of Gram negative and 29.8% of Gram positive infections were isolated from neutropenic patients, with* E. coli* and coagulase negative staphylococci being the most observed Gram negative and Gram positive microorganisms [16].

Blood stream infections were the major primary site of infection reported (43.75%) which has been documented in many studies cited before [1, 6, 9]. The etiology of infection varies according to the chemotherapy regimen used, sensitivity of microorganism, and the local climate [6].* Escherichia coli*,* Pseudomonas,* and* Klebsiella* are amongst the most frequently isolated microorganisms which is in accordance with the past literature. The Turkish also showed being* Escherichia coli* the most frequent among the isolated Gram negative organisms, constituting 58.4% of MDI [10].

In this study clinically documented infections (CDI) were 46.01% which is in accordance with the Brazilian data [11]. In 2003 we had 35% CDI and 15.7% MDI reported in 2006 [13, 14]. In 18.58% of infections no site of infection was found (PUO), it was reported as high as 47% and 28% in other studies [9, 11] and 60% reported in previous literature [13]. From previous local data PUO was reported as 15% in 2003 and 54.3% in 2006, respectively [13, 14]. This may reflect better yield and surveillance of cultures along with the advent of clinical and laboratory methods to document infections.

The median duration of neutropenia was 6 days while the median day of hospitalization was 7. It has been reported as 8.5 days from India and 9 days in Brazil [9, 11] in the past. It has been reported as 4–7 days in the literature. Fever was reported in 69.91% exhibiting the importance of this important clinical sign which is still in accordance with Brazilian and Indian studies referenced above.

The mortality rate reported here is 3% (7 patients) during hospital stay for febrile neutropenia treatment. Our cohort of patients was regarded as high risk as the commonest diagnosis was acute leukemia and they were on chemotherapy. The mortality has been variably reported as 5% to 39% in the above-described studies [9, 11, 12].

## 5. Conclusion

Febrile neutropenia is highly prevalent in the institutes involved in the treatment of hematological malignancies and is a major contributing factor in morbidity and mortality in postchemotherapy period. Gram negative infection with* Escherichia coli* is the most prevalent type of infection but showing considerable sensitivity to the current first-line antibiotic cover making this choice the most effective strategy; however, Gram positive infections showed significant decline as compared to previous antibiogram from same institute. We also suggest continuous surveillance of spectrum of locally prevalent pathogens and their susceptibility pattern which is essential in making local policies for empirical antibiotic treatment in febrile neutropenic patients.

## Figures and Tables

**Table 1:** Baseline characteristics (*n* = 226).

Baseline characteristics	*N*	%
Age, years		
mean	28.57	
Median	20	
Range	3–81	

Gender		
Male	173	76.5
Female	53	23.5

Signs and symptoms		
Fever	158	69.91
GI symptoms	73	32.3
Generalized weakness	69	30.5
Vertigo	3	1.3
Bleeding	38	16.81
Infection	3	1.32
Respiratory symptoms	31	27.43
Splenomegaly	16	7.1
Hepatomegaly	15	6.6

Clinical parameters at presentation		
HB, [g/dL, mean ± SD]	9.30 ± 1.96	
Range	2.6–14	
Mean platelet counts	56 ± 81.5	
Range	0–452	
ANC, [mean ± SD]	0.96 ± 1.79	

Hematological disorders documented		
ALL	91	41.3%
AML/MDS	50	22.1%
Aplastic anemia	32	14.2%
Lymphoma	19	8.4%
CML	13	5.8%
APML	11	4.7%
Others	10	4.42%

**Table 2:** Culture and sensitivity pattern of isolated microorganisms.

	PIP/TAZO	CEFO/SAL	CEFTAZI	CEFTRI	CARBEP	AMK	CIPRO	AMOX/CLAV	VANCO
Gram positive organism									
MSSA (2)	2 (100)	—	—	—	—	—	—	2 (100)	2 (100)
MRSA (4)	—	—	—	—	—	—	—	0 (0)	4 (100)
*Enterococcus* spp. (2)	2 (100)	0 (0)	0 (0)	0 (0)	0 (0)	—	—	1 (50)	2 (100)
*Strep.* spp. (2)	2 (100)	2 (100)	1 (50)	1 (50)	2 (100)	—	—	1 (50)	2 (100)
VRE (2)	0 (0)	0 (0)	0 (0)	0 (0)	0 (0)	—	—	0 (0)	0 (0)
Gram negative organism									
*E. coli* (27)	13 (48)	16 (96.2)	9 (33.3)	6 (22.2)	18 (66.6)	24 (88.8)	5 (18.5)	—	—
*K. pneumoniae* (12)	5 (41)	7 (58.3)	5 (41.6)	5 (41.6)	7 (58.3)	6 (50)	6 (50)	—	—
*Klebsiella* spp. (8)	7 (87.5)	8 (100)	4 (50)	5 (62.5)	6 (75)	7 (87.5)	7 (87.5)	—	—
*P. aeruginosa* (10)	10 (100)	9 (90)	10 (100)	8 (80)	7 (70)	10 (100)	9 (90)	—	—
*Pseudomonas* spp. (8)	6 (75)	7 (87.5)	5 (62.5)	3 (37.5)	4 (50)	4 (50)	6 (75)	—	—
*S. typhi* (3)	1 (33.3)	2 (66)	2 (66)	2 (66)	3 (100)	3 (100)	2 (66)	—	—
